# Associations between Feelings of Loneliness and Attitudes towards Physical Education in Contemporary Adolescents According to Sex, and Physical Activity Engagement

**DOI:** 10.3390/ijerph17155525

**Published:** 2020-07-30

**Authors:** Rosario Padial-Ruz, Gloria González-Campos, Félix Zurita-Ortega, M. Esther Puga-González

**Affiliations:** 1 Department of Didactics of Musical, Plastic and Corporal, University of Granada, 18011 Granada, Spain; rpadial@ugr.es (R.P.-R.); felixzo@ugr.es (F.Z.-O.); 2 Department of Human Motor Skills and Sport Performance, University of Sevilla, 41013 Sevilla, Spain; gloriagc@us.es; 3 Department of Didactics of Musical, Plastic and Corporal, University of Jaén, 2307 Jaen, Spain

**Keywords:** loneliness, attitude, adolescence, sex, physical activity, physical education

## Abstract

Background: Currently, adolescents grow up consuming a large amount of multimedia content and lead a sedentary lifestyle. As a result, emerging trends show greater feelings of loneliness. The present research seeks to describe adolescents’ attitudes towards Physical Education (PE), indices of loneliness and physical activity (PA) engagement. Further, it analyses associations between attitudes towards PE as a function of PA engagement and considers sex, loneliness and school year repetition as factors. Method: A sample of 2388 adolescents (43.3% males and 56.6% females) was recruited. Participants were aged 11–17 years (M = 13.8 years; SD = 1.2) and came from Spain. The following instruments were used: The Attitudes towards Physical Education questionnaire (AQPE) in order to analyze attitudes towards PE, and the University of California at Los Angeles (UCLA) loneliness scale to estimate loneliness. Results: With regard to sex and attitudes towards PE, significant differences were detected in relation to the difficulty of PE, with higher values being achieved in males (M = 2.4) than females (M = 2.3). A medium correlation emerged between the dimension of loneliness and the usefulness of PE (*r* = 0.323 **). Conclusions: It is confirmed that adolescents who experience greater levels of emotional or relational loneliness have less positive attitudes towards PE.

## 1. Introduction

Adolescents in the present day are more sedentary than those from previous generations [1]. Currently, both children and adolescents develop consuming multimedia content at unprecedented levels [2]. Young people currently report high indices of daily screen time in both developed countries and in societies with low socioeconomic levels [3]. In Western countries, average figures for time spent in front of the television, computer, or playing videogames, have reached as much as 3 to 6 h a day, depending on age and sex [4]. A consequence of this could be a negative impact on the psychological wellbeing of young people at a social level [5] as it leads to greater feelings of loneliness which inhibit interpersonal relationships. In representative samples of American adolescents aged between 13 and 18 years old, Twenge et al. [6] revealed a gradual decline in social interactions due to the excessive use of digital media. Amongst the different types of digital media, social networks particularly stand out as giving rise to a drastic increase in feelings of loneliness amongst young people.

Loneliness is defined as a situation in which an individual perceives a deficiency, both quantitatively and qualitatively, in their relationships [7]. According to Weiss [8], two types of loneliness are distinguished. Namely, these are emotional loneliness that is based on experiences of personal isolation and, social loneliness that results from a lack of integration and interpersonal relationships. Adolescence is the period during which individuals are most predisposed to poor emotional adjustment, with loneliness being one of the main issues characterising this stage [9].

Previously conducted studies have uncovered associations between loneliness and various variables such as sex [10,11], social networks [12] academic performance [13], etc. Further, Diehl et al. [14] indicate that a lack of physical activity (PA) engagement, among other things, is also related to a high index of social loneliness. These authors, therefore, suggest that programs should be delivered to promote PA or sport engagement as a protective factor against social isolation and feelings of loneliness.

With regard to sex and PA engagement, Werneck et al. [15] discovered in Brazilian adolescents that PA was associated with a lower prevalence of loneliness in males, while in females it was associated with a greater probability of having friends. As another issue to consider, individuals with physical disabilities show a greater predisposition towards developing stronger feelings of loneliness [16]. For this reason, it is necessary to implement PA programs specifically within this population, however, the most important aspect is that these programs must be adapted to the functional diversity of each adolescent.

Priesmeyer, Fedewa and Toland [17] concluded that PA promotion in schools and educational centers with children and adolescents is more of a need than an option. These authors argue that increasing engagement in physical exercise will contribute to creating a generation of individuals who are more emotionally healthy. In addition, it would even improve academic performance [18,19]. Studies indicate that a direct relationship is established between regular engagement in PA or sport and achievement of better academic performance [20].

From this, the importance of Physical Education (PE) becomes clear given that, among its main objectives, it aims to develop empathy. This plays a crucial role in encouraging prosocial dispositions of students, promoting the initiation and maintenance of positive relationships, widening contacts and associates, and reducing feelings of loneliness [21]. Nonetheless, Dowling [22] confirms that PE has little influence in the life of adolescents outside of school due to the increasing rate at which young people cease to engage in sport during puberty [23,24]. For this reason, some authors suggest moving away from the use of traditional sports models in PE classes, instead moving towards the promotion of satisfactory and fun experiences, which, in the future, reverse the decline in PA engagement seen in adolescence, both inside and outside of school [25,26].

Given the presented information, whether students have a favourable or unfavourable attitude towards PE will depend on multiple variables. This mainly refers to the fulfilment of the basic psychological needs of autonomy, perceived competence and social relatedness with peers during classes [27]. However, there also remains much to investigate in relation to what students think about PE and the feeling it gives them, with the aim of optimising teaching-learning processes in classes [28]. In this way, classes can contribute to the development of positive motivation and attitudes towards this discipline.

Thus, encouraging teachers to create a positive motivational climate which is based on feedback, different grouping systems, dynamic and varied tasks and adequate process evaluation, will favour the development of positive student attitudes towards PE [29]. Many authors recommend putting team-based sporting activities in place because they test the capacity of participants to cooperate, whilst also improving attitudes towards learning [30], in this way favouring interpersonal relationships. Koçak [31] demonstrates that cooperative learning can reduce loneliness. For this reason, PE classes may provide the perfect setting for adolescents to achieve good psychological wellbeing and a healthy lifestyle.

In consideration of the information presented above and based on the scarcity of studies examining the attitudes of adolescents towards PE classes, the following study is proposed that is based on various psychosocial-, academic- and PA-related aspects. The objective of the study was to describe and compare adolescents’ current attitudes towards PE, indices of loneliness, and PA engagement. A second objective was to analyse the existing relationships between attitudes towards PE as a function of whether or not individuals regularly engage in PA and whether sex and degree of loneliness are implied in this relationship.

## 2. Materials and Methods

### 2.1. Design and Participants

A total of 2388 adolescents participated in the present descriptive and cross-sectional study. Participants came from Spain and were aged between 11 and 17 years (M = 13.8 years; SD = 1.2), with 1036 (43.3%) being males and 1352 (56.6%) being females. All participants were undertaking compulsory secondary education. A process of convenience sampling was performed in order to select the sample. Inclusion criteria were defined by two tenets. The first was that students had to be enrolled in compulsory secondary education or the final year of primary education, and be fully able to independently engage in educational processes, and the second was that informed consent had to be received from parents or legal guardians. The sample was recruited from eight Spanish cities, with collaboration being requested from all the centres that voluntarily agreed to participate. It is necessary to indicate that 281 questionnaires were excluded after detecting that they had been incorrectly completed or had missing data. Sampling error was recorded as 0.04.

### 2.2. Variables and Instruments

- Ad-hoc self-completion form. Through this form, participants provided information about their sex (male or female), whether they had repeated any academic years (repeater or no repeater) and whether they engaged in more than 3 h a week of extracurricular physical activity (PA) (engages or does not engage) [18].
- Attitudes towards Physical Education questionnaire (AQPE). The original questionnaire developed by Moreno, Rodríguez and Gutiérrez [32] was used. The questionnaire is composed of 56 items which are rated along a four-point Likert type scale, where 1 = disagree, and 4 = totally agree. This instrument comprises seven different dimensions—evaluation of the Physical Education (PE) teacher and the subject itself, difficulty of PE, usefulness of PE, empathy with the teacher and the subject, agreement with subject organisation, preference for PE and sport, and PE as sport. Internal consistency of α = 0.75 was obtained for this instrument, this being acceptable and slightly higher than the value obtained by Moreno, Rodríguez and Gutiérrez [32] in the original study (α = 0.73).
- University of California at Los Angeles (UCLA) loneliness scale. An adaptation was used based on the original scale created by Russell, Peplau and Cutrona [33] and the version described by Russell [34]. The scale was adapted into Spanish by Expósito and Moya [35]. It contains 20 items and targets students from 11 years old and up. The original factorial structure is formed by a factor that provides a general index of perceived loneliness, alongside factors pertaining to different types of loneliness. On the one hand, emotional loneliness is found, composed of 11 items, whilst on the other hand, subjective evaluations of one’s social network are estimated through 9 items. When administering this instrument, various authors such as Buelga et al. [36] and Estévez, Murgui and Musitu [37] achieved Cronbach alphas greater than 0.80, while in the present study Cronbach alphas of 0.84 and 0.83, respectively (emotional and subjective scales), were obtained. The Cronbach alpha for the complete scale was 0.88.

### 2.3. Procedure

Educational centres were contacted by the University of Granada in order to inform them about the nature of the study, subsequently recruiting those centres that voluntarily agreed to participate in the research. Informed consent forms were then given out to students, requesting permission from parents or legal guardians. Following this, questionnaires were administered to groups during teaching hours. Anonymity of participant data was guaranteed, stating that collected data would be used only for scientific purposes. Researchers were present during data collection in order to guarantee correct implementation of study processes and resolve doubts. The present research received ethical approval from the ethical committee of the University of Granada, with the code641/CEIH/2018.

### 2.4. Data Analysis

Basic descriptive data were analysed using the statistical software *IBM SPSS^®^* in its version 24.0 (IBM Corp., Armonk, NY, USA) for *Windows*. The Kolgomorov–Smirnov test was used with the aim of analysing existing associations between the implied constructs and examine normality. For the statistical analysis of descriptive parameters, frequencies, percentages, means and standard deviations were used, where relevant, to describe all study variables. Similarly, Student’s t-test and bivariate correlations were used for the comparative analysis.

## 3. Results

A total of 2388 students participated in the conducted study, participants included both sexes (48.2% males and 51.8% females) and were completing the 3rd year of primary education or the 1st year of compulsory secondary education in one of the eight provinces of Andalusia. Of all participants, 87.2% had not repeated any academic year, while 12.8% had repeated at some point. Three-quarters (74%; *n* = 1768) of analysed students reported engaging in physical activity (PA), relative to almost a third who did not (26%; *n* = 620). When considering the dimension of attitudes towards PE, it can be seen from the values recorded that only the dimension relating to agreement with the organisation of PE has a value higher than three (M = 3.0). All remaining dimensions present values lower than this value, with the lowest mean pertaining to the usefulness of Physical Education (PE) (M = 2.0). In relation to the loneliness variable, the highest score is achieved in the dimension of subjective evaluation of the social network (M = 2.9), followed by general loneliness (M = 2.0) and, finally, emotional loneliness (M = 1.9) (Table 1).

With regard to the variable describing attitudes towards PE and its potential relationship with the five variables, as a function of sex and attitudes towards PA engagement (Table 2), statistically significant differences are detected in the case of PE difficulty (*p* = 0.000 ***; *** Statistically significant association between variables at the level 0.001). Further, higher values were reported by males (M = 2.4) for this variable, relative to females (M = 2.3). Differences are also detected in relation to the variable describing the usefulness of PE (*p* = 0.001 ***), with higher scores being reported by males (M = 2.0) than by females (M = 2.0). When the variable describing empathy with the teacher and subject is considered, a statistical association is again established (*p* = *0*.000 ***), with higher scores belonging to males (M = 2.5) in comparison to females (M = 2.3).

This same pattern is also detected in relation to preferences for physical activity and sport (*p* = 0.000 ***), with higher scores being reported by males (M = 2.3) than by females (M = 2.3). Finally, a statistically significant association is similarly found in relation to PE as a sport (*p* = *0*.020 ***), with higher values being provided by males (M = 2.4) than females (M = 2.4).

With regard to the dimensions of PA engagement and attitudes towards PE (Table 3), statistically significant differences were only shown in the dimension describing preference for PE and sport (*p* = *0*.002 ***). In relation to this, it can be observed that higher scores are reached by those who engage in PA (M = 2.3) than those who do not (M = 2.2). Differences are also detected (*p* = *0*.035 ***) in relation to the dimension describing empathy with the PE teacher and the subject itself, with higher mean scores belonging to those who engage in PA (M = 2.4), relative to those who do not (M = 2.3).

With regard to the dimensions describing whether participants had repeated an academic year and attitudes towards physical activity (Table 4), significance was achieved with respect to the usefulness of PE (*p* = *0*.002 ***), but not for the remaining dimensions (*p* > *0*.005). Usefulness of PE evaluations presented higher scores in those who had repeated (M = 2.1), relative to those who had not (M = 2.0).

Attending to the dimensions of loneliness in relation to attitudes towards PE (Table 5) (having previously analysed existing correlations between its dimensions), it is observed that when emotional loneliness increases, lower scores are given for the dimension pertaining to students’ ratings of the subject and the teacher who imparts it (*r* = −0.170 **; ** Correlation significant at the level of 0.05). In contrast, when emotional loneliness is greater, the subject is also perceived to be more difficult (*r* = 0.066 **).

In the same way, a medium correlation is produced between the dimensions of loneliness and the usefulness of PE (*r* = 0.323 **), however, when emotional isolation is greater, agreement with subject organisation decreases (*r* = −0.191 **). Of further interest, a small correlation was found concerning preferences for PE and sport (*r* = 0.107 **), and PE as sport (*r* = 0.085 **).Turning attention to subjective evaluations of one’s social network, it can be appreciated that when this is positive, so is the rating given to the subject and the teacher (*r* = 0.292 **). However, when this rating is higher, less usefulness is attributed to PE (*r* = −0.193 **).

In contrast, a correlation exists between subjective evaluations of one’s social network, and empathy towards the teacher and subject (r = 0.122 **). The same pattern occurs in relation to agreement with organisation of the subject (r = 0.243 **). At the same time, this relationship is clearly depicted when subjective evaluations of one’s social network are positive, as emotional loneliness is found to considerably decrease (*r* = −0.549 **).

With regard to the general dimension of loneliness, it is observed that when scores for this are high, ratings given to the subject and the teachers are lower. In contrast, a medium correlation exists which indicates that the larger the score pertaining to the loneliness dimension, the greater the score given for the usefulness of PE (*r* = 0.300 **). To a lesser extent, it can be stated that when general loneliness is greater, less empathy is given to the teacher and subject (*r* = −0.062 **). In a clearer way (*r* = −0.243 **), it is observed that when greater loneliness is reported, agreement with subject organisation is lower. Finally, it is highlighted, although only to a slight extent, that students who reported general indices of loneliness, also showed preferences for PE and sport (*r* = 0.067 **), and PE as sport (*r* = 0.070 **). To a much greater extent, it is also seen that a large correlation exists between general loneliness and emotional loneliness (*r* = 0.905 **).

## 4. Discussion

The results obtained are discussed below in consideration of the study objectives. Students selected for the sample were undertaking the 3rd year of primary education or the 1st year of compulsory secondary education (8–13 years). This educational period coincides with the beginning of adolescence, a stage characterised by various changes produced as a result of the transition from childhood to adulthood. It also common at this stage to experience significant emotional and behavioural problems, with loneliness at an emotional level standing out [38], alongside the abandonment of physical exercise, amongst other issues. Although students’ motivation towards physical activity (PA) is initially high in both sexes [39,40], diminishing interest and engagement is seen at increasing rates with advancing age [28,41]. Further, this decline is greater in females than in males. This is due to the fact that sex differences are magnified at this age [42,43], although authors such as Säfvenbom, Haugen and Bulie [44] argue that motivation is not determined by sex. Instead, they maintain that it is determined by individuals’ prior experiences with competitive sports, with males tending to engage in these sports more often.

Descriptive data obtained in the present research about PA engagement could initially seem optimistic given that they identify a large proportion of students declaring themselves to be physically active. Nonetheless, a certain meaningful percentage still remains of students who do not engage in PA and, above all, it must be kept in mind that this is just the beginning of the stage at which adolescents typically cease physical activity engagement.

In the descriptive data obtained about attitudes towards Physical Education (PE), students attributed high importance to the organization of the subject [45], with subject usefulness receiving the lowest scores. Thus, as age advances, an increasing lack of interest, and less favourable perceptions of subject relevance and importance are seen [41]. In exchange, contrasting data show high scores in relation to the usefulness of PE in the school setting and of PA in day-to-day life [46,47]. Students who consider the subject to be high in difficulty and to be well-organized, also show greater empathy to the PE teacher [45,48,49]. Nonetheless, this rating decreases when the usefulness of PE increases [50,51]. Along similar lines, previous studies produced evidence that leads us to believe that students maintain good attitudes towards PE when the aim of the activity focuses on enjoyment, fun and meaningful experiences. These qualities can be transferred to PA outside of the school setting [25,26] and may help satisfy the basic needs of competence, autonomy and relatedness [52].

With regard to students’ perceptions of loneliness, feelings of relational loneliness are reported more strongly than emotional loneliness. Affective traits are fundamental during adolescence [53]. Although the family factor is important at the start of this stage, as the stage progresses, family is relegated to a supporting role as peer relations take centre stage [54,55]. Indeed, it is fundamental to adolescents’ psychological wellbeing that they feel recognised by their peers, whilst also protecting them from isolation [36,56].

Another of the factors to cause negative effects, specifically in provoking feelings of relational loneliness and particularly amongst children, is excessive screen use [4,57]. Nonetheless, screen time can also be considered as a positive tool and necessary for the psychological and social wellbeing of disadvantaged populations [58]. This is even the case in emergency situations such as that currently being lived in with the new Coronavirus disease (COVID-19). Well guided use can have positive repercussions in young people as it favours virtual social relationships.

Results of the present study comparing attitudes towards PE, as a function of sex, indicate more positive attitudes amongst males [17,28]. Concretely, data pertaining to perceived usefulness of the subject, empathy with the teacher and the subject, preference for PA and sport, and PE as sport were significantly more positive among males. This collaborates other recent studies such as those conducted by Lago-Ballesteros, Navarro-Paton, Peixoto-Pino [59] and Padial-Ruz et al. [45]. Regrettably, these scores decrease with advancing age, with this change being even more evident in females [41,54]. It should be noted that in some other studies, differences were not found according to sex, or females even presented slightly more positive scores than males [44].

In contrast to data reported in other studies such as that conducted by Lago-Ballesteros et al. [59], results of the present research indicate greater perceptions of difficulty of PE classes among males. This is potentially because of the deterioration seen in their current motor skills [60], the scarce number of hours given to PE in educational centers, lack of PA engagement outside of the school setting and excessive screen time.

With regard to attitudes towards PE as a function of engagement in PA, we found a positive correlation between both. Students who regularly engage in moderate-to-vigorous intensity PA outside of the school timetable show greater self-efficacy, more favourable attitudes and greater enjoyment of PE, whilst also attributing PE with greater value and usefulness relative to those who do not engage in PA. This may explain why PE classes in the present day continue to deliver traditional content, related to sports that many adolescents engage in outside of school time. For this reason, students are likely to feel more competent when exposed to this content [44]. In contrast to the common consensus, Dowling [22] did not find any associations capable of predicting daily PA engagement in students.

In a similar sense, it is also seen that students who engage in PA present greater empathy towards the PE teacher and the subject itself. This is due to a favourable affective climate being created in which both the teacher and student operate, predicting positive attitudes towards PE [61,62]. In consideration of these data, other studies indicate that, with the passage of time, students show preferences for the teachers they feel closest to and have greatest personal empathy for, regardless of the material delivered [63].

Only one significant outcome was found in relation to attitudes towards PE as a function of whether the student had repeated an academic year or not. In this sense, children who had repeated a school year reported perceiving PE to be more useful than those who had never repeated. This could be motivated by a combination of factors. For instance, these students may find more enjoyment in PE classes [64]. It may also be relevant that PE is a practical subject, unlike other subjects [65], and that, through this, the needs of relatedness, autonomy and perceived competence are fulfilled [66].

In analysing the relationships established between PA and feelings of loneliness (emotional and relational), we found that physical inactivity emerges as a risk factor for loneliness in general, however, it is even more predictive of relational loneliness, with PA being an important protective factor [15]. Another risk factor, excessive screen use, can be added to this. This can negatively influence cognitive, affective-emotional and physical competencies, leading to feelings of loneliness and psychological issues such as stress, anxiety and depression [2,67].

With regard to this relationship, data obtained in the present study indicate that when students present higher levels of emotional loneliness, lower ratings are given of the PE teacher and subject itself [68,69] and organization of the subject [16], with the subject also being found to be more difficult [70]. These results could be related to the fact that adolescents who suffer from this type of loneliness present emotional maladjustment that has repercussions for their self-esteem and/or self-confidence. Further, these students feel that they lack the necessary integration to perform activities in this discipline. On the other hand, when the subject is perceived to be more difficult, stronger feelings of emotional loneliness are experienced. This is because the failure to achieve objectives makes these individuals feel frustration and a lack of competence [44]. This outcome appears not to be exclusive to the area of PE, but is a trait exhibited by these types of students towards all teachers and subjects in general [71]. Nonetheless, the children who participated in the present study who indicated greater emotional loneliness also showed a greater preference for the subject and considered it to be more useful. This coincides with results reported by Boekhout et al. [16], but is in contrast to other studies [72]. The reason for this may relate to the structure of the subject, in that students can see a way to improve physically, while also being able to participate in a dynamic and fun way with the rest of their classmates. Essentially, it is a practical subject with a different structure to other curricular materials and this likely explains these outcomes.

With regard to feelings of loneliness with respect to their social network, the present study indicates that the stronger the feeling of loneliness, the more highly valued the subject and its organisation, and the more the teacher is perceived to be empathetic with students. These data are probably due to the fact that adolescents consider the type of content and methods used in PE classes to be interesting and meaningful to them, and perceive a more favourable psychological climate to be established, encouraging greater relatedness than in other subjects.

Although emotional and social loneliness are considered to be independent given that they are predicted by different factors, a positive relationship exists between them. For this reason, children who indicated greater general loneliness also indicated emotional loneliness [15].

Strong interpersonal relationships, in other words, feeling connected to and supported by close others (close others being understood as family, the PE teacher, and classmates and friends), positively influences the attitudes and participation shown by children in PE classes and daily PA [73,74,75,76]. For this reason, perceived social support is an important protective factor which reduces general feelings of loneliness in adolescents [38] and is predictive of more positive attitudes towards PE.

The study presents some limitations, the first of which is related with its cross-sectional nature which impedes causal associations from being established. A second limitation is that the data cannot be generalised to the adult population. Another limitation of the present study relates to sample selection. Sampling was performed through a process of convenience sampling and this could introduce potential bias. Finally, the social context of the schools and the students, as well as the type of extracurricular PA practiced by both boys and girls, has not been taken into account, which would be important to take into account in future work.

## 5. Conclusions

As main conclusions, the present study shows that adolescents who feel high levels of emotional loneliness, perceive the subject of Physical Education (PE) to be more difficult, rate PE less positively and show less empathy towards their teachers. Further, the study reveals that adolescents who engage in more than 3 h a day of extracurricular physical activity (PA) report stronger preferences for PE and sport, and greater empathy towards PE and those who teach it. Thus, development of motivational strategies for PA and PE is urged. These should target family, friends and classmates, but above all the school itself. Curiously, adolescents who present higher general indices of loneliness rate PE as being more useful. Likewise, male adolescents report having more empathy with the subject of PE and its teachers, greater preferences for PA and sport, and recognise to a greater extent than females, the usefulness of PE for their vital development. Thus, in order to not intensify the sex differences seen in attitudes towards PE, it is suggested that professionals work on the relational competence of social interaction in classes. Given that females principally associate PA with a greater probability of making friends, such approaches are likely to succeed. It is also proposed that satisfactory and fun experiences should be created through PE, leading to the incorporation of PA in students’ daily lives.

As a point of interest, the present study reveals that students who repeat academic years attribute greater usefulness to their PE classes than those who do not repeat. Another interesting aspect is that the present research confirms that adolescents who positively rate social networks at a personal level present less emotional loneliness and more highly rate PE and PE teachers, although it is notable that they view it as being less useful. In this way, the present research opens up opportunities to further explore other significant variables in current adolescents as they relate to physical activity or sport engagement and feelings of loneliness. Indices of screen time should be considered, describing both frequency and modality (videogames, television, computer, mobile phone, etc.). Further, specific factors should be examined which impact children with regard to feelings of loneliness and attitudes towards PA, PE and sport. It would also be interesting to examine factors which influence social and relational loneliness in current adolescents, and uncover the most influential agents or figures in relation to this type of loneliness, focusing specifically on family, friends and classmates.

As a final conclusion, the social support perceived by students is highlighted from the perspective of a positive psychological climate in PE classes. In this sense, psychological climate is another predictor of favourable attitudes towards the subject and, as a consequence, a protective factor that reduces general feelings of loneliness in adolescents.

## Figures and Tables

**Table 1:** Descriptive statistics of the study variables.

Sex		Physical Activity Engagement	
Males	48.2% (*n* = 1151)	Yes	74% (*n* = 1768)
Females	51.8% (*n* = 1237)	No	26% (*n* = 620)
**Repeater**		**Attitude towards Physical Education** **M ± SD**	
Non repeater	87.2% (*n* = 2083)	Rating of the PE teacher and the subject itself	2.7 ± 0.521
Repeater	12.8% (*n* = 305)	Difficulty of PE	2.4 ± 0.546
**Loneliness Dimensions** **M/SD**		Usefulness of PE	2.0 ± 0.442
General loneliness	2.0 ± 0.505	Empathy with the teacher and the subject	2.4 ± 0.613
Subjective evaluation of one’s social network	2.9 ± 0.571	Agreement with subject organisation	3.0 ± 0.605
Emotional loneliness	1.9 ± 0.574	Preference for PE and sport	2.3 ± 0.618
		PE as sport	2.4 ± 0.680

Physical Education (PE); Mean (M); Standard Deviation (SD).

**Table 2:** Relation between attitudes towards physical education and sex.

Dimensions Sex		Mean	Standard Deviation	F	Sig
Rating of PE and the PE teacher	Males	2.7	0.516	0.125	0.724
	Females	2.7	0.526		
Difficulty of PE	Males	2.4	0.550	28.845	0.000 ***
	Females	2.3	0.535		
Usefulness of PE	Males	2.0	0.441	10.641	0.001 ***
	Females	2.0	0.440		
Empathy with the PE teacher and the subject itself	Males	2.5	0.611	25.808	0.000 ***
	Females	2.3	0.608		
Agreement with organisation of the subject	Males	3.0	0.587	0.083	0.774
	Females	3.0	0.620		
Preference for PE and sport	Males	2.3	0.617	22.515	0.000 ***
	Females	2.2	0.613		
PE as sport	Males	2.4	0.677	5.442	0.020 ***
	Females	2.4	0.681		

*** Statistically significant association between variables at the level 0.001. Physical Education (PE); Fisher test (F); Significance (Sig).

**Table 3:** Relation between dimensions of attitudes towards Physical Education according to FA engagement.

Attitude towards PEFA Engagement		Mean	Standard Deviation	F	Sig
Rating of the PE teacher and the subject itself	Engages	2.7	0.520	0.280	0.596
	Does not engage	2.6	0.519		
Difficulty of PE	Engages	2.4	0.550	1.248	0.264
	Does not engage	2.4	0.535		
Usefulness of PE	Engages	2.0	0.439	0.827	0.363
	Does not engage	2.0	0.447		
Empathy with the PE teacher and the subject itself	Engages	2.4	0.601	4.437	0.035 ***
	Does not engage	2.3	0.633		
Agreement with organisation of the subject	Engages	3.0	0.601	0.114	0.736
	Does not engage	2.9	0.608		
Preference for PE and sport	Engages	2.3	0.597	9.969	0.002 ***
	Does not engage	2.2	0.659		
PE as sport	Engages	2.4	0.675	0.136	0.712
	Does not engage	2.4	0.692		

*** Statistically significant association between variables at the level 0.001; Physical Education (PE); Physical Activity (PA); Fisher test (F); Significance (Sig).

**Table 4:** Relation between dimensions of repeater and attitudes towards Physical Education.

Dimensions	Rpt	Mean	Standard Deviation	F	Sig
Rating of the PE teacher and the subject itself	No	2.7	0.524	2.656	0.103
	Yes	2.6	0.202		
Difficulty of PE	No	2.4	0.543	3.798	0.051
	Yes	2.4	0.564		
Usefulness of PE	No	2.0	0.435	9.311	0.002 ***
	Yes	2.1	0.479		
Empathy with the PE teacher and the subject itself	No	2.4	0.607	0.373	0.541
	Yes	2.4	0.651		
Agreement with the organisation of the subject	No	3.0	0.600	1.308	0.253
	Yes	2.9	0.630		
Preference for PE and sport	No	2.3	0.617	0.121	0.728
	Yes	2.3	0.624		
PE as sport	No	2.4	0.675	1.872	0.171
	Yes	2.4	0.712		

*** Statistically significant association between variables at the level 0.001; Repeater (Rpt)_;_ Physical Education (PE); Fisher test (F); Significance (Sig).

**Table 5:** Dimensions of attitudes towards Physical Education and loneliness.

	RTPE	DPE	UPE	ETS	AOS	PPES	PES	EMLON	SESN	GL
RTPE	1									
DPE	0.212 **	1								
UPE	−0.133 **	0.253 **	1							
ETS	0.385 **	0.326 **	0.100 **	1						
AOS	0.334 **	0.121 **	−0.110 **	0.224 **	1					
PPES	0.251 **	0.216 **	0.250 **	0.376 **	0.071 **	1				
PES	0.106 **	0.285 **	0.253 **	0.229 **	0.074 **	0.233 **	1			
EMLON	−0.170 **	0.066 **	0.323 **	0.000	−0.191 **	0.107 **	0.085 **	1		
SESN	0.292 **	0.039	−0.193 **	0.122 **	0.243 **	−0.001	−0.033	−0.549 **	1	
GL	−0.255 **	0.021	0.300 **	−0.062 **	−0.243 **	0.067 **	0.070**	0.905 **	−0.852	1

** Correlation significant at the level of 0.05. Note: Rating of the PE teacher and subject (RTPE); difficulty of PE (DPE); usefulness of PE (UPE); empathy with the teacher and subject (ETS); agreement with the organisation of the subject (AOS); preference for PE and sport (PPES); PE as sport (PES); emotional loneliness (EMLON); subjective evaluation of social network (SESN); general loneliness (GL).
